# Effectiveness of Text Messaging Nudging to Increase Coverage of Influenza Vaccination Among Older Adults in Norway (InfluSMS Study): Protocol for a Randomized Controlled Trial

**DOI:** 10.2196/63938

**Published:** 2025-02-25

**Authors:** Bo T Hansen, Ole Klungsøyr, Angela S Labberton, Lauri Sääksvuori, Kjersti M Rydland, Liz E Ødeskaug, Torbjørn Wisløff, Hinta Meijerink

**Affiliations:** 1 Department of Infection Control and Vaccines Norwegian Institute of Public Health Oslo Norway; 2 Department of Research and Innovation Oslo Centre for Biostatistics and Epidemiology, Oslo University Hospital Oslo Norway; 3 Division of Health Services Norwegian Institute of Public Health Oslo Norway; 4 Centre for Health and Social Economics Finnish Institute for Health and Welfare Helsinki Finland; 5 INVEST Research Flagship Center University of Turku Turku Finland; 6 Department of Infection Control and Preparedness Norwegian Institute of Public Health Oslo Norway; 7 Health Services and Research Unit Akershus University Hospital Oslo Norway; 8 Institute of Clinical Medicine University of Oslo Oslo Norway

**Keywords:** influenza vaccination, coverage, uptake, behavioral nudging, vaccine hesitancy, randomized controlled trial, undervaccination, migrant health, mobile health, mHealth, smartphones, eHealth, SMS

## Abstract

**Background:**

The coverage of influenza vaccination among older adults in Norway is insufficient, especially in some immigrant groups. To improve public health, there is a need for an intervention that can increase influenza vaccination coverage. Further, interventions tailored to reduce potential barriers among immigrants can reduce health inequities.

**Objective:**

InfluSMS aims to determine if SMS nudging increases vaccination coverage among those aged 65 years or older (1) in Norway’s general population; (2) among immigrants born in Poland; and (3) among immigrants born in Ukraine; and evaluate the impact of SMS nudging in Norwegian versus in the official language of the native country of immigrants born in Poland or Ukraine.

**Methods:**

InfluSMS is a pragmatic randomized controlled trial conducted among people aged 65 years or older residing in Norway. Influenza vaccination coverage is the main outcome, measured in control and intervention arms for each of the 3 populations listed earlier. In all 3 populations, the control arm is standard care, that is, no individual reminder for influenza vaccination. All populations have an intervention arm that will receive an SMS nudge in the Norwegian language. In addition, the Polish and Ukrainian immigrant populations include a second intervention arm that will receive an SMS nudge in Polish or Ukrainian, respectively. In the general population, at least 23,485 individuals will be randomized to the SMS intervention arm while the rest of the population constitutes the control arm. In each of the 2 immigrant populations, we will randomize all eligible individuals 1:1:1 into the 3 arms. The intervention will take place at the start of the 2025-2026 influenza season. All eligible individuals will be passively followed up through the National Immunisation Registry, SYSVAK, from which individual influenza vaccination status 3 months after the SMS nudge will be collected. Coverage rates between arms within each population and effect sizes between the populations will be compared. The cost-effectiveness of SMS nudging will also be assessed.

**Results:**

The inclusion of participants will start in the third quarter of 2025, and the registry data will be available in the first quarter of 2026. Coverage rates of each strategy and coverage differences between strategies will be presented.

**Conclusions:**

SMS nudging is a scalable, inexpensive, and nonintrusive intervention that could be integrated into the national influenza vaccination program if the trial shows it effectively increases influenza vaccination coverage among older adults. Further, the trial will establish whether language is a barrier to influenza vaccination uptake among recent immigrant groups that have low influenza vaccination coverage, and to what extent this potential barrier can be diminished by SMS nudging in the official language of their native country.

**Trial Registration:**

ClinicalTrials.gov NCT06486766; https://clinicaltrials.gov/study/NCT06486766

**International Registered Report Identifier (IRRID):**

PRR1-10.2196/63938

## Introduction

### Background

Seasonal influenza has a high disease burden worldwide and may cause up to 5 million severe cases and 645,000 deaths annually [1]. Vaccination against influenza is generally safe and may be effective in reducing influenza-like illness and associated conditions [2-4]. It is a widely recommended public health intervention [5,6], especially for groups experiencing a disproportionately high influenza burden such as older adults, who have a higher risk of severe disease, hospitalization, and mortality [7,8]. However, influenza vaccination coverage generally falls far below the 75% target set by the World Health Organization [9], thus many older adults that could have benefitted from vaccination remain unprotected. Furthermore, vaccine hesitancy, defined as a “delay in acceptance or refusal of vaccination despite availability of vaccination services” [10], is widespread for influenza vaccination [11]. The World Health Organization has defined vaccine hesitancy as one of the 10 current threats to global health [12].

Several immigrant groups have shown a relatively low vaccination coverage in their adopted country and a relatively high burden of vaccine-preventable disease [13,14]. This may partly be related to differences in vaccine hesitancy, which may differ between countries [15,16]. It may also be associated with more limited access to vaccination among some immigrant groups, for instance, if immigrants do not understand the language in their adopted country. Whatever the reason, efforts are needed to narrow the gap in vaccination coverage and to promote public health as well as health equity. Tailored interventions addressing acceptability and access barriers faced by immigrants may be useful to achieve this end [14,17].

Vaccine hesitancy is a complex phenomenon that has several psychological components [18,19] that may be influenced by public health interventions that facilitate action and reduce barriers to uptake [20,21]. Behavioral nudges may improve compliance with the use of primary health care services [22], including influenza vaccination uptake among older adults [23]. However, the effectiveness of nudging varies substantially by mode, content, and population [24]. Nudging by reminding older adults to vaccinate against influenza has proven effective in increasing coverage but with far higher effectiveness for reminders sent by ordinary mail [25] than by a governmental electronic letter system [26]. SMS text message nudging may improve childhood vaccination [27], COVID-19 vaccination among health system employees [28], and influenza vaccination among patients due for a routine primary care visit [29]. However, a recent systematic review of randomized control trials (RCTs) on text messaging for improving vaccine uptake shows that very few trials have addressed influenza vaccination among older adults [30].

Norway’s influenza vaccination program recommends vaccination to people aged 65 years or older, as well as to other risk groups and health care personnel. Each municipality organizes local vaccination of risk groups. Vaccination is typically available at general practitioner’s offices, health clinics, and pharmacies, and is currently offered at an out-of-pocket cost ranging from NOK 150 to 500 (US $14 to $47). In general, vaccination at general practitioners requires the vaccinee to schedule an appointment, while vaccination at pharmacies can be drop-in or scheduled. There is no individual invitation, reminder, or scheduling for vaccination organized by the influenza vaccination program. COVID-19 vaccination is also recommended to the same age group at the same time of year. The same vaccination conditions apply to all residents of Norway, regardless of their citizenship or legal residency status.

InfluSMS is designed as a pragmatic, multiarmed, superiority RCT embedded in the Norwegian influenza vaccination program. In this trial, we aim to determine if sending a smartphone SMS nudge to be vaccinated against influenza can improve vaccination coverage in the general population aged 65 years or older in Norway, and further, to determine the impacts on vaccination coverage associated with providing an SMS nudge in the official language of the native countries of immigrants born in Poland or Ukraine. The Norwegian setting is well suited to conduct an RCT on SMS nudging among older adults, including in selected immigrant groups because there are nationwide administrative and health registries that can be used for this purpose.

An RCT on SMS nudging for influenza vaccination among older adults is warranted because the coverage is insufficient and because evidence on the effectiveness of this intervention is lacking [23,30]. Moreover, most older adults in Norway have a smartphone, thus an SMS intervention is likely to reach most of the eligible population. It is also a relatively nonintrusive and inexpensive intervention that is highly scalable, which thus could function as part of a national influenza vaccination program.

On average the influenza vaccination coverage among older individuals in Norway is 64%. However, the coverage among some immigrant groups is much lower. Immigrants born in Poland and Ukraine have especially low coverage rates with only 19% and 3% vaccinated in the 2023-2024 influenza season, respectively (Table 1). Poles and Ukrainians are the 2 largest immigrant groups in Norway. As of March 2024, there were 110,000 and 66,000 immigrants from Poland or Ukraine, respectively, accounting for 2.0% and 1.2%, of the entire Norwegian population. Even though the age profile of immigrants from Poland and Ukraine is younger than for some other country backgrounds, they still account for many older adult individuals (Table 1), and this demography is likely to increase in the coming years. Furthermore, Poles and Ukrainians are relatively recent immigrants and may as such have a limited proficiency in Norwegian. If this language barrier hinders their uptake of services, it could potentially be reduced by providing nudges in the official language of their native country.

**Table 1:** Nationwide coverage^a^ of influenza and COVID-19 vaccine among individuals aged 65 years or older^b^ during the 2023-2024 influenza season in Norway, stratified by country background^c^.

	Total population	Influenza vaccine^d^ , n (%)	COVID-19 vaccine^e^, n (%)
All	1,020,728	656,990 (64.4)	555,745 (54.4)
Born in Norway	941,657	623,598 (66.2)	530,923 (56.4)
Born in Sweden	7129	4520 (63.4)	3976 (55.8)
Born in Denmark	5966	3820 (64.0)	3372 (56.5)
Born in United Kingdom	4606	2888 (62.7)	2594 (56.3)
Born in Germany	3991	2000 (50.1)	1783 (44.7)
Born in Poland	3888	740 (19.0)	520 (13.4)
Born in Ukraine	3843	119 (3.1)	72 (1.9)

^a^Norwegian Immunisation Registry SYSVAK [31] data, extracted May 31, 2024.

^b^Age on December 31, 2023 (born 1958 or earlier).

^c^The most common country backgrounds among individuals aged 65 years or older.

^d^Received at least 1 dose of influenza vaccine between September 1, 2023, and May 29, 2024.

^e^Received at least 1 dose of COVID-19 vaccine between September 1, 2023, and May 29, 2024.

Poles and Ukrainians in Norway also had a very low uptake of the COVID-19 vaccine, both during [32] and after (Table 1) the pandemic, showing that low uptake of adult vaccines in these populations is not limited to influenza vaccination. Thus, interventions that may overcome vaccine hesitancy, which is prominent in Ukraine and Poland [15,16], as well as potential access barriers such as language, are likely to have a general relevance for vaccine uptake.

A recent systematic review highlighted language and tailored text message nudges for improving vaccine uptake as understudied [30]. We are not aware of any studies that have investigated the effectiveness of SMS nudging for influenza vaccination in several languages among immigrant populations.

### Objectives

The primary objectives of the InfluSMS trial are to establish whether (1) influenza vaccination nudging by SMS in the Norwegian language increases influenza vaccine coverage compared to standard care, among the Norwegian population aged 65 years and older; (2) influenza vaccination nudging by SMS in Polish or Ukrainian language increases influenza vaccine coverage compared to standard care, among older adults of Polish or Ukrainian background, respectively; (3) influenza vaccination nudging by SMS in Norwegian language increases influenza vaccine coverage compared to standard care, among older adults of Polish or Ukrainian background, respectively; and (4) influenza vaccination nudging by SMS in Polish or Ukrainian language increases influenza vaccine coverage more than nudging by SMS in Norwegian language among older adults of Polish or Ukrainian background, respectively.

For primary objectives 1-3, we hypothesize that SMS nudging will increase coverage. If this hypothesis is not confirmed, the interventions investigated here are futile from a public health perspective, and the remaining study objectives will not be investigated. For primary objective 4, we hypothesize that an SMS in the official language of the native country of each immigrant population will be more effective in increasing coverage than an SMS in Norwegian.

The secondary objectives are to establish whether (1) the effect of SMS nudging in the Norwegian language differs by country background; (2) the effect of SMS nudging in the official language of their native country differs between older adults of Polish or Ukrainian background; (3) SMS nudging for influenza vaccination is cost-effective compared to standard care; (4) SMS nudging for influenza vaccination influences coverage of COVID-19 vaccination; and (5) the time to vaccination differs by the comparisons described in the primary objectives.

Secondary objectives 1-4 are exploratory and have no hypotheses. For secondary objective 5, we hypothesize that SMS nudging will shorten the time elapsed to vaccination.

## Methods

### Participants

#### Overview

All Norwegian residents have a unique personal identification number, which is used in health and administrative national registries and can be used to merge data across different registries. In this study, we identify all eligible individuals based on information available from the National Population Register and the Norwegian Immunisation Registry SYSVAK.

The trial has 3 separate populations of individuals who are aged 65 years or older and who reside in Norway, namely (1) the entire Norwegian population (except immigrants born in Poland or Ukraine); (2) immigrants born in Poland; and (3) immigrants born in Ukraine.

#### Inclusion Criteria

- Aged 65 years and older (age at the end of 2025, ie, born in 1960 or earlier).
- Resident in Norway and have a valid ID number on September 1, 2025.
- Have a smartphone number in the common contact register.

#### Exclusion Criteria

- Have received the 2025 influenza vaccine prior to the SMS nudge dispatch date.
- Have emigrated or died before SMS nudge dispatch date.

### Interventions

**Figure 1:**
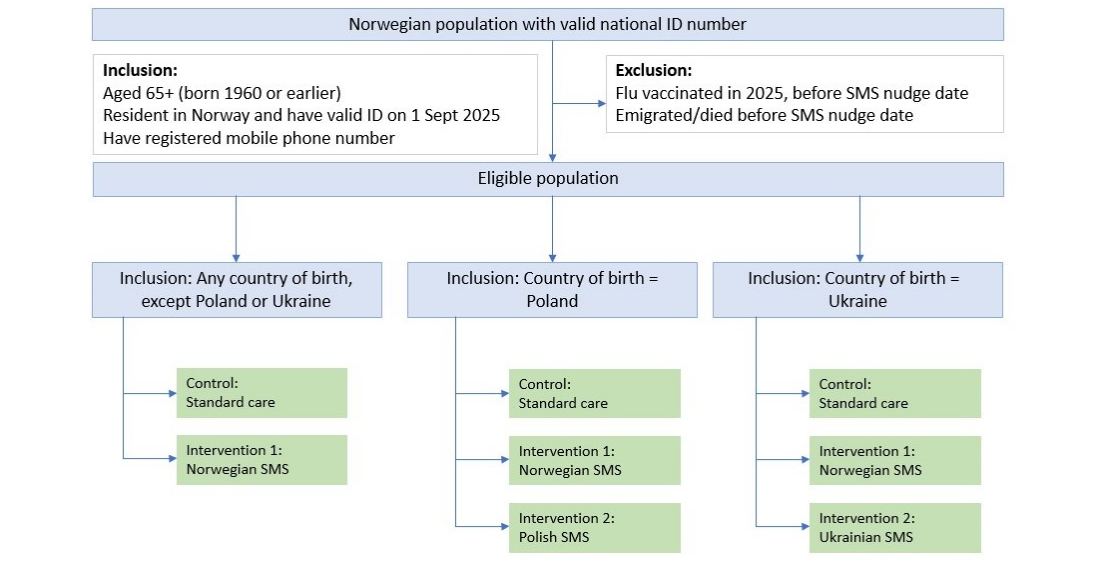
InfluSMS participant flowchart.

Participants in the intervention arms receive an SMS reminder for influenza vaccination in Norwegian, Polish, or Ukrainian. Participants in the control arm do not receive an SMS reminder for influenza vaccination, which is “standard care” in Norway. A description and overview of the InfluSMS study arms and participant flow are shown in Table 2 and Figure 1.

**Table 2:** InfluSMS study arms.

Study arm	Description	Population
Control	Standard care: Individuals do not receive an SMS reminder for influenza vaccination.	General population Immigrants born in Poland Immigrants born in Ukraine
Intervention 1	Norwegian nudge: Individuals receive an SMS in Norwegian language at the start of the influenza season; to remind them they are recommended to get the influenza vaccine	General population Immigrants born in Poland Immigrants born in Ukraine
Intervention 2	Polish or Ukrainian nudge: Individuals receive an SMS in the official language of their native country (ie, Polish or Ukrainian) at the start of the influenza season, to remind them they are recommended to get the influenza vaccine	Immigrants born in Poland Immigrants born in Ukraine

### Outcomes

The primary outcome measure is having been vaccinated against influenza during the 3 months following the SMS dispatch date in the 2025-2026 influenza season (yes or no, for each individual), as registered in the Norwegian Immunisation Registry SYSVAK [31]. Notification of influenza vaccination to SYSVAK is obligatory for vaccine administrators and does not require patient consent. Follow-up during this trial is passive and does not require any effort from the participants.

Effects will be assessed by influenza vaccination coverage, that is, percent uptake, which will be compared between strategies. SMS nudging will take place at the earliest timepoint at which vaccines for the upcoming influenza season become widely available across the country, which usually is around week 42. All SMS nudges will be dispatched within 1 week. Each individual will be followed up through SYSVAK for 3 months after their SMS dispatch date. We end follow-up at 3 months because we expect that the potential effect of an SMS nudge will be exhausted by then, and because most vaccinations in the influenza vaccination program will have occurred by the end of this period.

Time to influenza vaccination, that is, the number of days elapsed between the SMS dispatch date and the influenza vaccination date (as registered in SYSVAK), is a secondary outcome measure.

We will also address coverage of COVID-19 vaccination as an outcome in the exploratory analyses.

### Sample Size

#### Primary Objective 1: General Population in Norway

We will identify all individuals in the National Population Register that fulfill the eligibility criteria, and among those, make a random selection to receive the Norwegian nudge (intervention 1). We power for a 1% point minimal detectable increase in uptake in the SMS arm compared to the control arm in the general population because the intervention is relatively inexpensive and might be cost-effective even with low effectiveness on coverage. To achieve this minimal detectable increase with a baseline uptake rate of 60%, a power of 0.8, and a 1-sided α of .05, we need to randomize 18,788 individuals to intervention 1. However, based on available statistics, some 20% of individuals may have received the influenza vaccine before the scheduled trial start date and will be excluded from the trial after randomization. We thus need to randomly sample at least 23,485 individuals aged 65 years or older from the entire Norwegian population (except those born in Poland or Ukraine) to be allocated to intervention 1 to be able to detect an increase of 1 percentage point (23,485×80/100=18,788). The control group will be the rest of the Norwegian population that fulfills the eligibility criteria. Currently, there are circa 1.1 million persons aged 65 years or older who reside in Norway. We use this large group as a control because it gives the study high power, the inclusion of individuals receiving standard care carries no cost, and it will provide real-world data on the entire older adult population in Norway.

#### Primary Objectives 2-4: Immigrants Born in Poland or Ukraine

To maximize power among individuals born in Poland or Ukraine, we will include all eligible individuals and randomly assign them 1:1:1 to each of the 3 study arms. The number we will be able to include in the trial will most likely be higher in 2025 than in 2023 (Table 1) due to the age structure in these immigrant populations and due to new migration. Based on current national demographic statistics, we anticipate there will be some 6000 individuals aged 65 years or older from each immigrant group at the start of the trial. However, some 10% may be excluded due to not having a registered phone number, and a further 1% and 6% (Table 1) may be excluded due to influenza vaccination before the scheduled SMS dispatch date among the Ukrainian and Polish immigrant populations, respectively. Thus, we expect to be able to randomize 5346 Ukrainian immigrants, giving 1782 individuals in each study arm. With a baseline uptake rate of 3% (Table 1), a power of 0.8, and a 1-sided α of .05, this gives a minimal detectable coverage increase of 2 percentage points, adjusted for 3 pairwise comparisons. Similarly, we expect to be able to randomize 5076 Polish immigrants, giving 1692 individuals in each study arm. With a baseline uptake rate of 19% (Table 1), a power of 0.8, and a 1-sided α of .05, this gives a minimal detectable coverage increase of 4% points, adjusted for 3 pairwise comparisons.

In conclusion, the minimal detectable coverage increases of this trial fall within the range of effect sizes reported for other modes of nudging investigated in neighboring countries that share a similar influenza vaccination policy. Specifically, a recent study from Denmark reported that nudging by electronic letters increased coverage by approximately 1% points [26], while a recent study from Finland reported that nudging by ordinary mail on average increased coverage by approximately 6% points [25].

#### Stratified Analyses

We will also perform analyses stratified by sex, broad categories of age, years of residency in Norway, and household composition. These analyses will have lower power to detect the differences indicated earlier because the sample size in each stratum will be lower than for the overall population.

### Recruitment

The study uses data from all residents in Norway who meet the eligibility criteria. The data is retrieved from complete nationwide registries that contain routinely collected information. Therefore, recruitment and follow-up are entirely registry-based and do not require any action by the participants.

### Assignment of Interventions

Assignment of treatment arm (control, intervention 1, and intervention 2) will be randomized, automated, and concealed. The identities of the participants will not be decipherable by the data managers.

We will use an external service provider to identify the eligible study population, perform the random selection and randomization procedures, and dispatch the SMS. The provider will be selected through a tendering process. All data management by the service provider will be done in close collaboration with and under the supervision of the project group. Data management, random selection, and randomization procedures will be performed with reproducible code that includes documented and adequate software functions, preferentially in R programming (R Foundation for Statistical Computing). For the Norwegian population, individuals in intervention arm 1 will be randomly selected from the whole eligible population, while the remainder of the eligible population will serve as the control arm. Similarly, we will use complete random assignment to treatment arm for the Polish and Ukrainian populations. Examples of R functions that serve these purposes are sample_n (from the *dplyr* package) and complete_ra (from the *randomizr* package). Random selection and randomization procedures will be performed after the assessment of the exclusion criteria. The project group will inspect the randomization code before dispatching the SMS.

### Blinding

The outcome measure of the trial is based solely on routinely collected data and those reporting or registering this information will not be aware of whether a vaccinated individual has received an SMS nudge. Thus, the trial is blinded to the outcomes assessor. Moreover, treatment allocation is randomized and computer-generated, there is no interaction between the InfluSMS project team and study participants, and the InfluSMS researchers work with deidentified data and do not know the identity of any participant in the dataset. Due to the inherent characteristics of the intervention, treatment allocation cannot be blinded for participants.

### Data Collection

**Figure 2:**
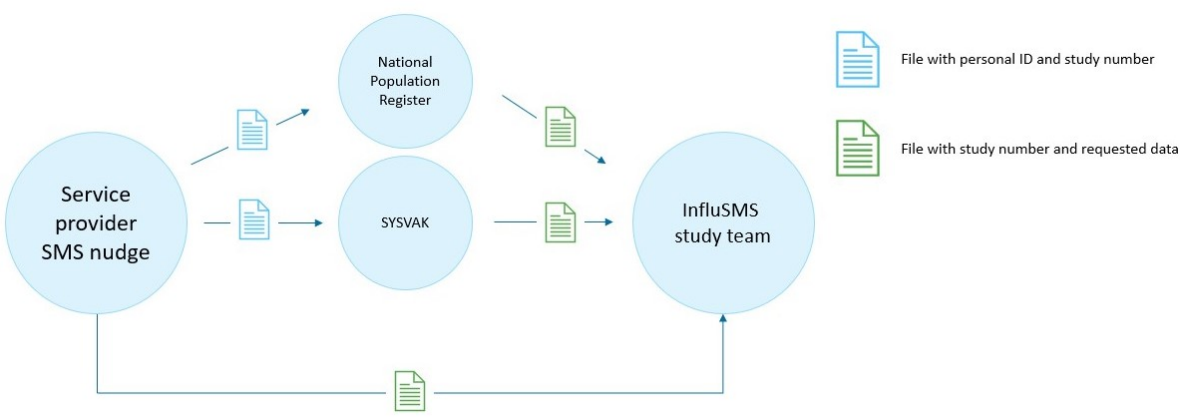
InfluSMS data flow and deidentification of registry data.

The external service provider will be asked to prepare a key file that includes all individuals born in 1960 or earlier in the Norwegian population, with associated individual information including the individual national ID number, the household ID, a randomly generated study ID unique for each individual, and categorical variables indicating treatment arm and exclusion status. This key file will be shared with the National Population Register and SYSVAK. All registries involved will merge requested variables from their registry to this file by the national ID number, and forward the resulting file, excluding the national ID number, to the InfluSMS team. Using the study ID, InfluSMS researchers will then merge the deidentified files from the registries. The key linking the national ID numbers and the study ID numbers will be kept by the service provider and will never be accessible to the InfluSMS team. The procedure is illustrated in Figure 2.

Table 3 lists the variables that will be used in the trial. Note that the national ID will only be used to generate the dataset.

**Table 3:** List of variables collected or generated in the trial.

Variable		Reason for inclusion
**National Population Register**		
	National ID	Used to identify eligible individuals and link to study ID. Not shared with InfluSMS project group
	Household composition	Variable identifying whether eligible individuals live in the same household, to enable sensitivity analyses
	Year of birth	Potential confounder (age)
	Sex	Potential confounder
	Vital status, with date	Exclusion of individuals who died or emigrated
	Country of birth	Determine if born in Poland or Ukraine
	Immigration date	Determine time living in Norway (potential confounder)
**SYSVAK**		
	National ID	Used to identify eligible individuals and link to study ID. Not shared with InfluSMS project group
	Date of vaccination	Determine if date of vaccine was before/after nudge, and impact of vaccination
	Disease against which the vaccine protects: Influenza COVID-19	Determine who received each vaccine, to calculate vaccination coverages
**External service provider**		
	National ID	Used to identify eligible individuals and link to study ID. Not shared with InfluSMS project group
	Demography: General population Born in Poland Born in Ukraine	Study populations
	Randomized, and received intervention (SMS): Yes No	Identify control or intervention groups
	Date SMS was sent	Start of intervention
	SMS language: Norwegian Polish Ukrainian	Type of intervention
	Excluded from study: No Yes, with code for reason	Quantify exclusions
	SMS sending errors: Yes: error in sending No: no known error	Quantify errors with intervention or infrastructure

### Data Management

All data management and analyses will be scripted in R code so that each step can be reproduced and quality assured. Range and other logical data checks will be performed for quality assurance. Programming will be validated by several team members. Data and scripts will be used and stored in a high-security data zone that will be accessible only to team members via login.

### Statistical Methods

All individuals randomized will be analyzed, except individuals who die or emigrate during follow-up, who will be excluded from the analyses. We expect exclusions to be evenly distributed between treatment arms due to the randomized design. Additionally, we anticipate a low proportion of excluded individuals given the relatively short follow-up time of the trial. According to official statistics [33], we can expect approximately 2800 deaths and 100 emigrations per month among the nearly 1.1 million persons aged 65 years or older residing in Norway, which accounts for 0.3% of this population.

As recommended for binary RCT outcomes [34], coverage differences between treatment arms will be expressed on absolute and relative scales, that is, as percentage point differences and as relative risks, with associated 95% Wald CIs. The primary focus will be on absolute scale analyses because they may be most informative for public health decision-making. Absolute estimates will be derived from linear regressions [35], while relative estimates will be derived from generalized linear models with a log-binomial link function [36]. Overall interactions (specifically secondary objectives 1 and 2, where differences between treatment arms will be compared between country backgrounds) will be assessed by likelihood ratio tests and, if significant, further analyzed by the participation differences between factor levels of the variables that interact.

We will compare the time to vaccination (ie, the number of days elapsed between the SMS dispatch date and the influenza vaccination date) between trial arms by the Kaplan-Meier method and the log-rank test.

We assume the registry data for randomized individuals to be close to complete and thus will not impute missing values.

As sensitivity analyses, we will exclude households consisting of at least two or more people aged 65 or older. Comparing the results of these analyses with the main results will indicate whether there may be spillover effects of the SMS interventions within households. Moreover, it will exclude persons living in nursing homes, who may experience easier access and less autonomy regarding influenza vaccination. In exploratory analyses, we will also address the potential effects of SMS nudging for influenza vaccination on the coverage of COVID-19 vaccination. We will use the same statistical methods for these sensitivity and exploratory analyses as described for the main analyses.

We will develop a decision analytic model that can evaluate the potential cost-utility of each nudge compared to standard care and each other. The model will be based on coverage differences observed in the trial and will incorporate implications of increased coverage on the spread of influenza and its consequences for the use of health care resources. Data on reduced influenza from increased uptake will be based on effect estimates of vaccination from the best available scientific evidence. Costs associated with the intervention as well as with increased uptake of the vaccine will be recorded in detail during the project phase. Direct medical costs related to the downstream use of health care services will also be incorporated into the model. The cost-utility analysis will be based on Norwegian guidelines [37], conducted according to standard principles for health economic evaluation [38], and reported according to current guidelines [39].

### Data Monitoring

The trial does not pose any harm to participants; thus, the project does not have a data monitoring committee or plans for auditing of trial conduct. Similarly, there will be no interim analyses before the end date of follow-up and no stopping guidelines for the trial.

### Ethical Considerations

We have submitted the project for initial evaluation to the Regional Committees for Medical and Health Research Ethics, who recently determined that the project does not fall under the jurisdiction of the Health Research Act [40] (reference #787321). We will thus apply for exemption from the duty of confidentiality from the Health Directorate. The rationale for this decision was that the objective of the research is not to gain new knowledge on health and disease as such, with reference to paragraphs 2 and 4a of the Health Research Act. The application was considered by the REK sør-øst B committee.

This study cannot be performed based on informed consent. Exemption from the duty of confidentiality gives a permit to access and merge the personal registry data without obtaining informed consent. We have conducted several similar registry-based studies. It is our experience that exemption for the duty of confidentiality is granted for studies of this nature, that have large potential public health benefits and no direct consequence for participants [41-44]. The registries own their data and will grant the project data access based on the permits we supply in our data application (ie, exemption from the duty of confidentiality).

Smartphone numbers will be accessed through the Contact and Reservation Register, in which every Norwegian citizen who has logged into a Norwegian public service using an electronic ID is registered. Individuals can opt out of the register, and those who have done so will not receive an SMS as part of this study. About 95% of the adult Norwegian population is part of the Contact and Reservation Register. Opting out will not be considered in the analyses in this pragmatic trial because access to smartphone numbers would be through the same register if SMS reminders were part of the influenza vaccination program. We expect that the drop-out rate will be similar across treatment arms due to the randomized design.

We will submit the protocol and approvals together with a dedicated data protection impact assessment for assessment by the data protection officer at the National Institute of Public Health. The data protection officer oversees data processing in research projects to ensure it is in accordance with applicable data protection laws. The main principles regarding data protection and confidentiality in the project are described in the Data Collection and Data Management sections.

In case of protocol amendments, all stakeholders that have granted approvals to the project, as well as Clinicaltrials.gov, will be notified. None of the project members have any financial or other conflicting interests to declare.

Data access is contingent on the specific permits described above. Thus, data are only accessible to project members. To advise the project, users of Polish, Ukrainian, and Norwegian origin will be part of the project group. We will also have focus groups with users from each nationality to help formulate acceptable and adequate SMS nudges.

### Dissemination

We plan to publish the protocol and the findings of the trial in international peer-reviewed scientific journals. Authorship will be granted according to the Vancouver Convention. Professional writers will not be used.

The findings will also be presented at national and international conferences. To inform the public, we will publish information about the trial on a project web page. Information aimed at the public will mainly be in Norwegian. Further, we will present the project to various stakeholder groups, notably the main user groups of the study (organizations for older adults and immigrant groups) and public health organizations.

## Results

As of July 3, 2024, the project has received funding for protocol development from Foundation Dam (grant SDAM_FOR-558766) and we are in the process of obtaining permits, agreements, and access to the infrastructure needed to conduct the trial. Inclusion of participants will start in the third quarter of 2025 and the outcome registry data will be available in the first quarter of 2026. We plan to publish the results during the fourth quarter of 2026.

The results will be reported according to the Consolidated Standards of Reporting Trials (CONSORT) statement on eHealth [45,46]. We will present the baseline characteristics of the 3 study populations, and describe the coverage by treatment arm and population, for strata of age, sex, and duration of residency. We will present the absolute and relative coverage differences between the treatment arms as described in objectives 1-4. In addition, we present comparisons of effect sizes between populations as described in secondary objectives 1 and 2, on absolute and relative scales. The primary focus will be on absolute scale analyses for the coverage analyses (primary outcome measure). We also compare the time to vaccination between treatment arms (the secondary outcome measure). All effects are presented with 95% CIs. We also present the likelihood ratio test *P* value for interaction terms. Table shells for the analyses described earlier can be found in Multimedia Appendix 1. The main findings may also be illustrated in the figures.

## Discussion

This pragmatic RCT will provide insights into the effectiveness of SMS reminders in improving influenza vaccination coverage among older adults, which was specifically mentioned as a knowledge gap in a recent review on this topic [23]. The trial will estimate the effectiveness in the older adult population in Norway at large, and among older adult immigrants from Poland or Ukraine, which are large immigrant populations that have a particularly low influenza vaccination coverage in Norway. For the immigrant populations, the trial will also address whether the language of their adopted country may represent a barrier to influenza vaccination uptake that can be reduced by SMS nudging in the official language of their native country. If SMS nudging is effective, it is a strategy that may be feasible for implementation in a nationwide influenza vaccination program because it is scalable, inexpensive, and non-intrusive. Since influenza vaccination protects against influenza and its sequelae, a higher influenza vaccination coverage will benefit society through lower health care costs, as well as the individuals who get vaccinated through direct protection against influenza.

Strengths of this trial include the use of data on nearly all individuals aged 65 years or older residing in Norway, whose health and administrative data are captured in nationwide registries without any loss to follow-up. This eliminates selection and response biases associated with surveys, ensures high quality of the main outcome measure of the trial, that is, influenza vaccination coverage, and yields results that are valid at the national level. Further characteristics that contribute to the high internal validity of this study include the randomized design, no experimenter bias, and standardized procedures for all participants. This trial also has high external validity since it is conducted in real life and will thus yield data that will be entirely representative of how these interventions would work should they become part of an adult vaccination program. Moreover, the results are likely to be of relevance internationally, both as a proof of concept of whether SMS interventions might be effective in increasing adult vaccination coverage and more directly for the immigrant groups addressed here, which likely experience similarly low coverage, for similar reasons, in other adopted countries. The trial will also establish the feasibility of implementing an SMS nudging intervention using the infrastructure available in Norway, for instance in terms of timely access to the necessary health and administrative registry data. Novelty is another strength of the study since we are not aware of other trials with similar interventions and objectives. The trial will also be informed by users, which may enhance its relevance to the populations targeted by the interventions.

The trial also has several limitations, most of which relate to the number of Polish and Ukrainian immigrants we will be able to randomize. First, the number of persons in the immigrant groups who are aged 65 years or older in the 2025-2026 influenza season is a moving target that will be influenced by the influx and outflux of people by that time, as well as the exact underlying age distribution of these immigrant populations. Second, our estimates of the fraction of people missing a registered smartphone number are based on statistics for the whole Norwegian population, and we do not know whether this fraction may be different for the Polish and Ukrainian sub-populations. Third, the minimum detectable effect size for the Polish and Ukrainian populations is approximately 4% and 2% points, respectively. This may be a limitation because even smaller effect sizes might be of public health interest for an inexpensive intervention. Finally, even though we will initiate the trial at the very start of the influenza season, some people will already have been vaccinated by this point, and they will be excluded from participation in the trial. The number of excluded individuals will depend on national vaccine distribution logistics and the epidemiology of influenza in the trial season, which are beyond our control.

The current influenza vaccination program lacks an individual cue to action, which could potentially be more effective than a general recommendation that lacks personalization or direct communication. Sending SMS nudges for influenza vaccination is one way to make individual contact that is likely to reach a very large proportion of the eligible population. If sending SMS reminders is effective, even small effect sizes may benefit public health and be cost-effective. Furthermore, nudges tailored to immigrant groups that have very low uptake of influenza vaccine may reduce inequalities in the use of this preventive health care service and, thus, in health. Insights from the interventions investigated in the current trial can extend to other vaccination contexts and be of international relevance.

## Appendix

Multimedia Appendix 1 Table shells for statistical analyses.

## Abbreviations

CONSORT: Consolidated Standards of Reporting Trials
RCT: randomized controlled trial

## Data Availability

The datasets generated in this study are not publicly available because the participants have not consented to it. The data are available from the corresponding author upon reasonable request with permission from the relevant Norwegian authorities.

## References

[1] Iuliano AD, Roguski KM, Chang HH, Muscatello DJ, Palekar R, Tempia S, Cohen C, Gran JM, Schanzer D, Cowling BJ, Wu P, Kyncl J, Ang LW, Park M, Redlberger-Fritz M, Yu H, Espenhain L, Krishnan A, Emukule G, van Asten L, Pereira da Silva S, Aungkulanon S, Buchholz U, Widdowson M, Bresee JS (2018). Estimates of global seasonal influenza-associated respiratory mortality: a modelling study. Lancet.

[2] Rondy M, El Omeiri N, Thompson MG, Levêque A, Moren A, Sullivan SG (2017). Effectiveness of influenza vaccines in preventing severe influenza illness among adults: a systematic review and meta-analysis of test-negative design case-control studies. J Infect.

[3] Demicheli V, Jefferson T, Di Pietrantonj C, Ferroni E, Thorning S, Thomas RE, Rivetti A (2018). Vaccines for preventing influenza in the elderly. Cochrane Database Syst Rev.

[4] Demicheli V, Jefferson T, Ferroni E, Rivetti A, Di Pietrantonj C (2018). Vaccines for preventing influenza in healthy adults. Cochrane Database Syst Rev.

[5] (2022). Vaccines against influenza: WHO position paper—May 2022. World Health Organization.

[6] Immunization Agenda 2030: a global strategy to leave no one behind. World Health Organization.

[7] GBD 2017 Influenza Collaborators (2019). Mortality, morbidity, and hospitalisations due to influenza lower respiratory tract infections, 2017: an analysis for the Global Burden of Disease Study 2017. Lancet Respir Med.

[8] Mertz D, Kim TH, Johnstone J, Lam P, Science M, Kuster SP, Fadel SA, Tran D, Fernandez E, Bhatnagar N, Loeb M (2013). Populations at risk for severe or complicated influenza illness: systematic review and meta-analysis. BMJ.

[9] Jorgensen P, Mereckiene J, Cotter S, Johansen K, Tsolova S, Brown C (2018). How close are countries of the WHO European region to achieving the goal of vaccinating 75% of key risk groups against influenza? Results from national surveys on seasonal influenza vaccination programmes, 2008/2009 to 2014/2015. Vaccine.

[10] MacDonald NE, SAGE Working Group on Vaccine Hesitancy (2015). Vaccine hesitancy: definition, scope and determinants. Vaccine.

[11] Schmid P, Rauber D, Betsch C, Lidolt G, Denker ML (2017). Barriers of influenza vaccination intention and behavior—a systematic review of influenza vaccine hesitancy, 2005-2016. PLoS One.

[12] (2019). Ten threats to global health in 2019. World Health Organization.

[13] Charania NA, Gaze N, Kung JY, Brooks S (2019). Vaccine-preventable diseases and immunisation coverage among migrants and non-migrants worldwide: a scoping review of published literature, 2006 to 2016. Vaccine.

[14] Crawshaw AF, Farah Y, Deal A, Rustage K, Hayward SE, Carter J, Knights F, Goldsmith LP, Campos-Matos I, Wurie F, Majeed A, Bedford H, Forster AS, Hargreaves S (2022). Defining the determinants of vaccine uptake and undervaccination in migrant populations in Europe to improve routine and COVID-19 vaccine uptake: a systematic review. Lancet Infect Dis.

[15] Larson HJ, de Figueiredo A, Xiahong Z, Schulz WS, Verger P, Johnston IG, Cook AR, Jones NS (2016). The state of vaccine confidence 2016: global insights through a 67-country survey. EBioMedicine.

[16] de Figueiredo A, Simas C, Karafillakis E, Paterson P, Larson HJ (2020). Mapping global trends in vaccine confidence and investigating barriers to vaccine uptake: a large-scale retrospective temporal modelling study. Lancet.

[17] Charania NA, Gaze N, Kung JY, Brooks S (2020). Interventions to reduce the burden of vaccine-preventable diseases among migrants and refugees worldwide: a scoping review of published literature, 2006-2018. Vaccine.

[18] Thomson A, Robinson K, Vallée-Tourangeau G (2016). The 5As: a practical taxonomy for the determinants of vaccine uptake. Vaccine.

[19] Geiger M, Rees F, Lilleholt L, Santana AP, Zettler I, Wilhelm O, Betsch C, Böhm R (2022). Measuring the 7Cs of vaccination readiness. Eur J Psychol Assess.

[20] Brewer NT, Chapman GB, Rothman AJ, Leask J, Kempe A (2017). Increasing vaccination: putting psychological science into action. Psychol Sci Public Interest.

[21] Malik AA, Ahmed N, Shafiq M, Elharake JA, James E, Nyhan K, Paintsil E, Melchinger HC, Team YBI, Malik FA, Omer SB (2023). Behavioral interventions for vaccination uptake: a systematic review and meta-analysis. Health Policy.

[22] Schwebel FJ, Larimer ME (2018). Using text message reminders in health care services: a narrative literature review. Internet Interv.

[23] Thomas RE, Lorenzetti DL (2018). Interventions to increase influenza vaccination rates of those 60 years and older in the community. Cochrane Database Syst Rev.

[24] Reñosa MDC, Landicho J, Wachinger J, Dalglish SL, Bärnighausen K, Bärnighausen T, McMahon SA (2021). Nudging toward vaccination: a systematic review. BMJ Glob Health.

[25] Sääksvuori L, Betsch C, Nohynek H, Salo H, Sivelä J, Böhm R (2022). Information nudges for influenza vaccination: evidence from a large-scale cluster-randomized controlled trial in Finland. PLoS Med.

[26] Johansen ND, Vaduganathan M, Bhatt AS, Lee SG, Modin D, Claggett BL, Dueger EL, Samson SI, Loiacono MM, Køber L, Solomon SD, Sivapalan P, Jensen JUS, Martel CJ, Valentiner-Branth P, Krause TG, Biering-Sørensen Tor (2023). Electronic nudges to increase influenza vaccination uptake in Denmark: a nationwide, pragmatic, registry-based, randomised implementation trial. Lancet.

[27] Currie GE, McLeod C, Waddington C, Snelling TL (2024). SMS-based interventions for improving child and adolescent vaccine coverage and timeliness: a systematic review. BMC Public Health.

[28] Patel MS, Fogel R, Winegar AL, Horseman C, Ottenbacher A, Habash S, Dukes JL, Brinson TC, Price SC, Masoudi FA, Cacchione J, Yehia BR (2022). Effect of text message reminders and vaccine reservations on adherence to a health system COVID-19 vaccination policy: a randomized clinical trial. JAMA Netw Open.

[29] Milkman KL, Patel MS, Gandhi L, Graci H, Gromet D, Ho QDH, Kay J, Lee T, Akinola M, Beshears J, Bogard J, Buttenheim A, Chabris C, Chapman GB, Choi JJ, Dai H, Fox CR, Goren A, Hilchey M, Hmurovic J, John L, Karlan D, Kim M, Laibson DI, Lamberton C, Madrian BC, Meyer MN, Modanu M, Nam J, Rogers T, Rondina R, Saccardo S, Shermohammed M, Soman D, Sparks J, Warren C, Weber M, Berman R, Evans C, Snider C, Tsukayama E, Van den Bulte C, Volpp K, Duckworth A (2021). A megastudy of text-based nudges encouraging patients to get vaccinated at an upcoming doctor's appointment. Proc Natl Acad Sci U S A.

[30] Louw GE, Hohlfeld AS, Kalan R, Engel ME (2024). Mobile phone text message reminders to improve vaccination uptake: a systematic review and meta-analysis. Vaccines.

[31] Trogstad L, Ung G, Hagerup-Jenssen M, Cappelen I, Haugen I, Feiring B (2012). The Norwegian immunisation register—SYSVAK. Euro Surveill.

[32] Kraft KB, Godøy AA, Vinjerui KH, Kour P, Kjøllesdal MKR, Indseth T (2022). COVID-19 vaccination coverage by immigrant background. Tidsskr Nor Laegeforen.

[33] (2023). StatBank Norway.

[34] Schulz KF, Altman DG, Moher D, CONSORT Group (2010). CONSORT 2010 statement: updated guidelines for reporting parallel group randomized trials. Ann Intern Med.

[35] Hellevik O (2007). Linear versus logistic regression when the dependent variable is a dichotomy. Qual Quant.

[36] McCullagh P, Nelder JA (1989). Generalized Linear Models.

[37] Guidelines for the submission of documentation for single technology assessment (STA) of pharmaceuticals. Norwegian Medical Products Agency.

[38] Drummond MFS, Sculpher MJ, Torrance GW, O'Brien BJ, Stoddart GL (2015). Methods for the Economic Evaluation of Health Care Programmes.

[39] Husereau D, Drummond M, Augustovski F, de Bekker-Grob E, Briggs AH, Carswell C, Caulley L, Chaiyakunapruk N, Greenberg D, Loder E, Mauskopf J, Mullins CD, Petrou S, Pwu R, Staniszewska S (2022). Consolidated Health Economic Evaluation Reporting Standards 2022 (CHEERS 2022) statement: updated reporting guidance for health economic evaluations. MDM Policy Pract.

[40] (2008). ACT 2008-06-20 no. 44: Act on Medical and Health Research (the Health Research Act). Ministry of Health and Care Services.

[41] Hansen BT, Campbell S, Burger E, Nygård M (2015). Correlates of HPV vaccine uptake in school-based routine vaccination of preadolescent girls in Norway: a register-based study of 90,000 girls and their parents. Prev Med.

[42] Hansen BT, Nygård M, Castle PE, Burger EA, Aasbø G (2024). Sociodemographic characteristics associated with cervical cancer screening participation by send-to-all and opt-in HPV self-sampling: who benefits? Results from a randomized controlled trial among long-term non-attending women in Norway. Int J Cancer.

[43] Meijerink H, Shelil M, Jani-Bølstad J, Dvergsdal ET, Madslien EH, Wilberg M, Gundersen RB, Sæbø JI, Thorseng AA, Iversen BG (2024). Does integration with national registers improve the data completeness of local COVID-19 contact tracing tools? A register-based study in Norway, May 2020-September 2021. BMC Health Serv Res.

[44] Seppälä E, Dahl J, Veneti L, Rydland KM, Klüwer B, Rohringer A, Meijerink H (2024). Covid-19 and influenza vaccine effectiveness against associated hospital admission and death among individuals over 65 years in Norway: a population-based cohort study, 3 October 2022 to 20 June 2023. Vaccine.

[45] Altman DG (1996). Better reporting of randomised controlled trials: the CONSORT statement. BMJ.

[46] Eysenbach G, CONSORT-EHEALTH Group (2011). CONSORT-EHEALTH: improving and standardizing evaluation reports of web-based and mobile health interventions. J Med Internet Res.

