# Supplementary material for: Effectiveness of Text Messaging Nudging to Increase Coverage of Influenza Vaccination Among Older Adults in Norway (InfluSMS Study): Protocol for a Randomized Controlled Trial
Source: JMIR Res Protoc. 2025 Feb 25;14:e63938. doi: 10.2196/63938 (PMC11897661; doi:10.2196/63938)
Supplement: Multimedia Appendix 1 [file resprot_v14i1e63938_app1.docx]

**Appendix, “Effectiveness of SMS nudging to increase coverage of influenza vaccination among older adults in Norway: Protocol for a randomized controlled trial (InfluSMS study)”**

**Table shells**

| **Table shell 1: Baseline characteristics of the study populations (N, %)** | | | | | | | | | |
| --- | --- | --- | --- | --- | --- | --- | --- | --- | --- |
|  |  | Norway | | Polish immigrants | | | Ukrainian immigrants | | |
|  |  | Control (no SMS) | Intervention1  (Norwegian SMS) | Control (no SMS) | Intervention1 (Norwegian SMS) | Intervention2 (Polish SMS) | Control (no SMS) | Intervention1 (Norwegian SMS) | Intervention2 (Ukrainian SMS) |
| Total |  |  |  |  |  |  |  |  |  |
| Sex | Female |  |  |  |  |  |  |  |  |
|  | Male |  |  |  |  |  |  |  |  |
| Age group* | 65-69 |  |  |  |  |  |  |  |  |
|  | 70-74 |  |  |  |  |  |  |  |  |
|  | 75-79 |  |  |  |  |  |  |  |  |
|  | 80+ |  |  |  |  |  |  |  |  |
| Residency* | <1 years | - | - |  |  |  |  |  |  |
|  | 1–5 years | - | - |  |  |  |  |  |  |
|  | 6-10 years | - | - |  |  |  |  |  |  |
|  | ≥11 years | - | - |  |  |  |  |  |  |
| Region* | North |  |  |  |  |  |  |  |  |
|  | West |  |  |  |  |  |  |  |  |
|  | South |  |  |  |  |  |  |  |  |
|  | Mid |  |  |  |  |  |  |  |  |
|  | East |  |  |  |  |  |  |  |  |

*Categories are suggestive, and subject to change based on the data

| **Table shell 2: Influenza vaccine coverage by treatment arm and by study population** | | | | | | |
| --- | --- | --- | --- | --- | --- | --- |
|  | Control (no SMS) | | Intervention1  (Norwegian SMS) | | Intervention2  (Polish/Ukrainian SMS) | |
|  | Vaccinated | | Vaccinated | | Vaccinated | |
|  | N | % | N | % | N | % |
| Total |  |  |  |  |  |  |
| Norway (total) |  |  |  |  | - | - |
| By age groups |  |  |  |  | - | - |
| By sex |  |  |  |  | - | - |
| Poland (total) |  |  |  |  |  |  |
| By age groups |  |  |  |  |  |  |
| By sex |  |  |  |  |  |  |
| By residency |  |  |  |  |  |  |
| Ukraine (total) |  |  |  |  |  |  |
| By age groups |  |  |  |  |  |  |
| By sex |  |  |  |  |  |  |
| By residency |  |  |  |  |  |  |

| **Table shell 3. Coverage differences between treatment arms, by study population** | | | | | | |
| --- | --- | --- | --- | --- | --- | --- |
|  | Control vs. Intervention 1  (Objectives 1 and 3) | | Control vs. Intervention 2  (Objective 2) | | Intervention 1 vs. Intervention 2  (Objective 4) | |
|  | ACD  (95% CI) | RCD  (95% CI) | ACD  (95% CI) | RCD  (95% CI) | ACD  (95% CI) | RCD  (95% CI) |
| Norway (total) |  |  | - | - | - | - |
| By age groups |  |  | - | - | - | - |
| By sex |  |  | - | - | - | - |
| Poland (total) |  |  |  |  |  |  |
| By age groups |  |  |  |  |  |  |
| By sex |  |  |  |  |  |  |
| By residency |  |  |  |  |  |  |
| Ukraine (total) |  |  |  |  |  |  |
| By age groups |  |  |  |  |  |  |
| By sex |  |  |  |  |  |  |
| By residency |  |  |  |  |  |  |

ACD = absolute coverage difference

RCD = relative coverage difference

| **Table shell 4. Absolute coverage differences between treatment arms and between study populations** | | | | | | |
| --- | --- | --- | --- | --- | --- | --- |
|  | Control vs. Intervention 1 (Objective 5) | | | Control vs. Intervention 2 (Objective 6) | | |
|  | ACD  (95%CI) | Difference in ACD (95% CI) | P interaction | ACD  (95% CI) | Difference in ACD (95% CI) | P Interaction |
| Norway |  | (Reference) |  | - | - |  |
| Poland |  |  |  |  | (Reference) |  |
| Ukraine |  |  |  |  |  |  |

ACD = absolute coverage difference

| **Table shell 5. Relative coverage differences between treatment arms and between study populations** | | | | | | |
| --- | --- | --- | --- | --- | --- | --- |
|  | Control vs. Intervention 1 (Objective 5) | | | Control vs. Intervention 2 (Objective 6) | | |
|  | RCD  (95%CI) | Difference in RCD (95% CI) | P interaction | RCD  (95% CI) | Difference in RCD (95% CI) | P Interaction |
| Norway |  | 1 (reference) |  | - | - |  |
| Poland |  |  |  |  | 1 (reference) |  |
| Ukraine |  |  |  |  |  |  |

RCD = relative coverage difference

| **Table shell 6. Number of days to influenza vaccination by treatment arm and by study population (Objective 9)*** | | | | | | |
| --- | --- | --- | --- | --- | --- | --- |
|  | Control (no SMS) | | Intervention1  (Norwegian SMS) | | Intervention2  (Polish/Ukrainian SMS) | |
|  | Median # days  (95% CI) | | Median # days  (95% CI) | | Median # days  (95% CI) | |
| Norway |  |  |  |  |  | - |
| Poland |  |  |  |  |  |  |
| Ukraine |  |  |  |  |  |  |

*Corresponding Kaplan-Meier curves and log-rank tests will be presented for the primary objective comparisons
